# Heptacarbonyl-1κ^3^ *C*,2κ^4^ *C*-(4-phenyl­pyridine-1κ*N*)di-μ-phenyltellurido-1:2κ^4^ *Te*:*Te*-dirhenium(I)

**DOI:** 10.1107/S1600536810012389

**Published:** 2010-04-14

**Authors:** A. Vanitha, J. Muthukumaran, R. Krishna, Bala Manimaran

**Affiliations:** a Department of Chemistry, Pondicherry University, Puducherry 605 014, India; b Centre for Bioinformatics, Pondicherry University, Puducherry 605 014, India

## Abstract

In the title complex, [Re_2_(C_6_H_5_Te)_2_(C_11_H_9_N)(CO)_7_], two Re atoms are coordinated in slightly distorted octa­hedral coordination environments and are bridged by two Te atoms, which are coordinated in trigonal-pyramidal environments. The torsion angle for the Te—Re—Te—Re sequence of atoms is 17.06 (3)°. The crystal structure is stabilized by weak C—H⋯O and C—H⋯π inter­actions. In addition, there are Te⋯Te distances [4.0392 (12) Å] and O⋯O distances [2.902 (19) Å] which are shorter than the sum of the van der Waals radii for these atoms. A short inter­molecular lone pair⋯π distance [C O⋯*Cg* = 3.31 (2) Å] is also observed.

## Related literature

For the biological applications of Re and Te compounds, see: Begum *et al.* (2008 ▶); Atwood *et al.* (1983 ▶); Zhang & Leong (2000 ▶); Lima *et al.* (2009 ▶); Cunha *et al.* (2009 ▶); Kopf-Maier & Klapötke (1992 ▶). For a related structure, see Cecconi *et al.* (1998 ▶). For an example of a structure with weak Te⋯Te contacts, see: Ritch & Chivers (2009 ▶). For details of electron lone pair inter­actions, see: Jain *et al.* (2009 ▶).
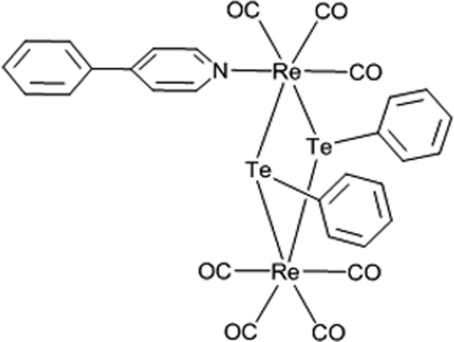


## Experimental

### 

#### Crystal data

[Re_2_(C_6_H_5_Te)_2_(C_11_H_9_N)(CO)_7_]
*M* *_r_* = 1133.06
Monoclinic, 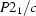

*a* = 18.549 (2) Å
*b* = 12.3624 (12) Å
*c* = 13.7768 (11) Å
β = 92.927 (9)°
*V* = 3155.0 (5) Å^3^
*Z* = 4
Mo *K*α radiation
μ = 9.53 mm^−1^
*T* = 150 K
0.23 × 0.18 × 0.15 mm

#### Data collection

Oxford Diffraction Xcalibur-S diffractometer
Absorption correction: multi-scan (*CrysAlis PRO*; Oxford Diffraction, 2009 ▶) *T*_min_ = 0.218, *T*_max_ = 0.329
22729 measured reflections
5553 independent reflections
4237 reflections with *I* > 2σ(*I*)
*R*_int_ = 0.098

#### Refinement

*R*[*F* ^2^ > 2σ(*F* ^2^)] = 0.058
*wR*(*F* ^2^) = 0.135
*S* = 1.03
5553 reflections
379 parameters
12 restraints
H-atom parameters constrained
Δρ_max_ = 2.25 e Å^−3^
Δρ_min_ = −3.27 e Å^−3^

### 

Data collection: *CrysAlis CCD* (Oxford Diffraction, 2009 ▶); cell refinement: *CrysAlis RED* (Oxford Diffraction, 2009 ▶); data reduction: *CrysAlis RED*; program(s) used to solve structure: *SHELXS97* (Sheldrick, 2008 ▶); program(s) used to refine structure: *SHELXL97* (Sheldrick, 2008 ▶); molecular graphics: *ORTEP-3 for Windows* (Farrugia, 1997 ▶) and *PLATON* (Spek, 2009 ▶); software used to prepare material for publication: *PLATON*.

## Supplementary Material

Crystal structure: contains datablocks I, global. DOI: 10.1107/S1600536810012389/lh5009sup1.cif

Structure factors: contains datablocks I. DOI: 10.1107/S1600536810012389/lh5009Isup2.hkl

Additional supplementary materials:  crystallographic information; 3D view; checkCIF report

## Figures and Tables

**Table 1:** Hydrogen-bond geometry (Å, °) *Cg* is the centroid of the C14–C19 ring.

*D*—H⋯*A*	*D*—H	H⋯*A*	*D*⋯*A*	*D*—H⋯*A*
C16—H16⋯O4^i^	0.95	2.55	3.33 (2)	139
C11—H11⋯O4^ii^	0.95	2.71	3.160 (18)	110
C17—H17⋯O1^iii^	0.95	2.46	3.271 (19)	144
C26—H26⋯O2^iv^	0.95	2.71	3.34 (2)	124
C21—H21⋯O3^v^	0.95	2.45	3.200 (16)	136
C29—H29⋯*Cg*^vi^	0.95	2.79	3.378 (15)	121

Symmetry codes: (i) 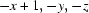; (ii) 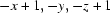; (iii) 
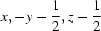
; (iv) 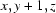; (v) 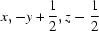; (vi) 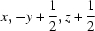.

**Fig. 1.**
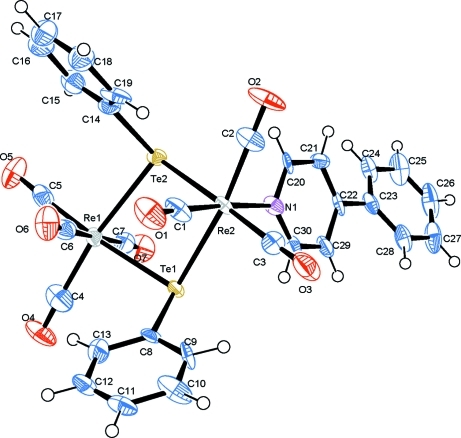
: The molecular structure of title compound, showing the atom-labeling scheme. Displacement ellipsoids are drawn at the 50% probability level.

**Fig. 2.**
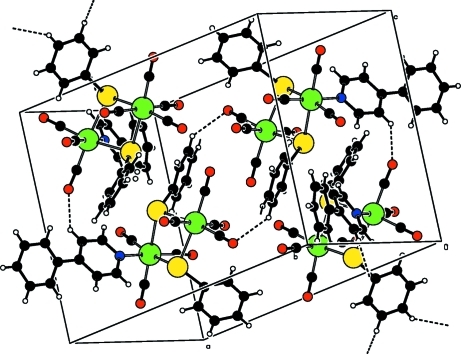
: Part of the crystal structure of the title compound showing weak C-H···O interactions as dashed lines.

## supplementary crystallographic information

### Comment

Rhenium and tellurium compounds posses interesting and promising biological applications (Begum *et al.*, 2008; Atwood *et al.*,1983; Zhang & Leong, 2000). Organic telluranes have been used as protease inhibitors and have applications in a wide range of disease models. Some tellurium derivatives also exhibit an antioxidant as well as immunomodulatory effects (Cunha *et al.*, 2009). Recently, a novel organotellurium compound RT-01 was proven to act as antileishmanial agent for the disease Leishmaniasis (Lima *et al.*, 2009). Rhenium containing compounds are found to have anti tumor and cytostatic activity (Kopf-Maier & Klapotke, 1992). Considering the importance of the compounds, we have synthesized and undertaken the single crystal structure determination of title compound of which the molecular structure is shown in Fig. 1.

The Re_2_Te_2_ four-membered ring of atoms deviates significantly from planarity as described by the Te2-Re1-Te1-Re2 torsion angle of 17.06 (3)°. The Re-Te distances in the title compound can be compared with those in a related structure (Cecconi *et al.*, 1998). An Intermolecular O2···O2(-x, -y, -z) = 2.902 (19)Å distance is less than the sum of the van der Waals radii for these atoms as is a short intermolecular Te···Te distance [Te1···Te2(x, 0.5-y, 0.5+z) = 4.0392 (12) Å]. The Te···Te contacts were also noticed in a Te structure reported by Ritch & Chivers (2009). A lone pair···.π interaction as described by Jain, *et al.*, 2009 is also evident with O3···Cg(C15-C28) = 3.31 (2) Å. Furthemore the crystal structure is stabilzed by weak C-H···O (Fig 2.) and C—H···π interactions.

### Experimental

A mixture of Re_2_(CO)_10_ (130 mg, 0.2 mmol) and diphenyl ditelluride (41 mg, 0.1 mmol), 4-phenylpyridine (93 mg, 0.6 mmol) were taken in a 50 ml two neck Schlenk flask and fitted with a reflux condenser. The system was evacuated and purged with N_2_. Freshly distilled mesitylene (30 ml) was added under N_2_ atmosphere. The reaction mixture was heated to 403K under N_2_ for 6 h and allowed to cool to room temperature. The mesitylene was removed by vacuum distillation and the solid mixture was washed with hexane, chromatographed on silicagel using dichloromethane and hexane as eluent to obtain white color solid of mono substituted [Phpy(CO)_3_Re(µ-TeC_6_H_5_)_2_Re(CO)_4_] (61 mg, 27% (based on Re_2_(CO)_10_) compound. Single crystals of the title compound were obtained by slow diffusion of hexane into a concentrated solution of the title compound in dichloromethane at 278K.

### Refinement

The hydrogen atoms were placed in calculated positions (C-H = 0.95Å) and included in the refinement in riding-model approximation  with U_iso_(H) = 1.2U_eq_(C). Restraints were applied to the anisotropic displacement parameters of atoms C3 and C18 using the ISOR command in SHELXL-97 (Sheldrick, 2008). The need to restrain these parameters and the presence of some larger positive and lower negative density peaks in the difference Fourier may indicate the lowered precision of the structure.

### Crystal data

**Table:**

[Re_2_(C_6_H_5_Te)_2_(C_11_H_9_N)(CO)_7_]	*F*(000) = 2064
*M**_r_* = 1133.06	*D*_x_ = 2.385 Mg m^−^^3^
Monoclinic, *P*2_1_/*c*	Mo *K*α radiation, λ = 0.71073 Å
Hall symbol: -P 2ybc	Cell parameters from 4169 reflections
*a* = 18.549 (2) Å	θ = 3.0–32.8°
*b* = 12.3624 (12) Å	µ = 9.53 mm^−^^1^
*c* = 13.7768 (11) Å	*T* = 150 K
β = 92.927 (9)°	Plate, yellow
*V* = 3155.0 (5) Å^3^	0.23 × 0.18 × 0.15 mm
*Z* = 4	

### Data collection

**Table:**

Oxford Diffraction Xcalibur-S diffractometer	5553 independent reflections
Radiation source: fine-focus sealed tube	4237 reflections with *I* > 2σ(*I*)
graphite	*R*_int_ = 0.098
Detector resolution: 0 pixels mm^-1^	θ_max_ = 25.0°, θ_min_ = 3.0°
ω scans	*h* = −22→22
Absorption correction: multi-scan (*CrysAlis PRO*; Oxford Diffraction, 2009)	*k* = −14→14
*T*_min_ = 0.218, *T*_max_ = 0.329	*l* = −16→16
22729 measured reflections	

### Refinement

**Table:**

Refinement on *F*^2^	Primary atom site location: structure-invariant direct methods
Least-squares matrix: full	Secondary atom site location: difference Fourier map
*R*[*F*^2^ > 2σ(*F*^2^)] = 0.058	Hydrogen site location: inferred from neighbouring sites
*wR*(*F*^2^) = 0.135	H-atom parameters constrained
*S* = 1.02	*w* = 1/[σ^2^(*F*_o_^2^) + (0.0776*P*)^2^] where *P* = (*F*_o_^2^ + 2*F*_c_^2^)/3
5553 reflections	(Δ/σ)_max_ < 0.001
379 parameters	Δρ_max_ = 2.25 e Å^−^^3^
12 restraints	Δρ_min_ = −3.27 e Å^−^^3^

### Special details

**Table:**

Experimental. Mean plane calculation for molecule m1 = -0.11114(0.00018) m2 = 0.99346(0.00000) m3 = -0.02622(0.00031) D = 1.07228(0.00135) Atom d s d/s (d/s)**2 Re1 * -0.1293 0.0005 -256.403 65742.445 Te1 * 0.2845 0.0008 379.024 143659.391 Re2 * -0.1290 0.0005 -255.731 65398.523 Te2 * 0.2873 0.0008 382.870 146589.250 ============ Sum((d/s)**2) for starred atoms 421389.625 Chi-squared at 95% for 1 degrees of freedom: 3.84 The group of atoms deviates significantly from planarity
Geometry. All esds (except the esd in the dihedral angle between two l.s. planes) are estimated using the full covariance matrix. The cell esds are taken into account individually in the estimation of esds in distances, angles and torsion angles; correlations between esds in cell parameters are only used when they are defined by crystal symmetry. An approximate (isotropic) treatment of cell esds is used for estimating esds involving l.s. planes.
Refinement. Refinement of *F*^2^ against ALL reflections. The weighted *R*-factor wR and goodness of fit *S* are based on *F*^2^, conventional *R*-factors *R* are based on *F*, with *F* set to zero for negative *F*^2^. The threshold expression of *F*^2^ > σ(*F*^2^) is used only for calculating *R*-factors(gt) etc. and is not relevant to the choice of reflections for refinement. *R*-factors based on *F*^2^ are statistically about twice as large as those based on *F*, and *R*- factors based on ALL data will be even larger.

### Fractional atomic coordinates and isotropic or equivalent isotropic displacement parameters (Å^2^)

**Table:**

	*x*	*y*	*z*	*U*_iso_*/*U*_eq_	
Re1	0.40209 (3)	0.14784 (4)	0.15533 (4)	0.02636 (17)	
Re2	0.18223 (3)	0.11194 (4)	0.19775 (4)	0.02389 (16)	
Te1	0.30828 (5)	0.16925 (6)	0.30588 (6)	0.0238 (2)	
Te2	0.27148 (5)	0.15735 (6)	0.04687 (6)	0.0243 (2)	
O1	0.2230 (6)	−0.1266 (8)	0.2217 (9)	0.054 (3)	
O2	0.0593 (6)	0.0574 (9)	0.0498 (8)	0.059 (3)	
O3	0.0858 (6)	0.0750 (11)	0.3700 (8)	0.059 (3)	
O4	0.5334 (7)	0.1542 (11)	0.2999 (10)	0.070 (4)	
O5	0.4940 (7)	0.1321 (9)	−0.0267 (9)	0.061 (3)	
O6	0.3823 (6)	−0.1045 (8)	0.1626 (9)	0.050 (3)	
O7	0.4133 (7)	0.3982 (8)	0.1504 (9)	0.056 (3)	
N1	0.1553 (6)	0.2867 (8)	0.1910 (8)	0.027 (2)	
C1	0.2085 (8)	−0.0355 (12)	0.2112 (10)	0.037 (4)	
C2	0.1057 (8)	0.0777 (10)	0.1053 (10)	0.035 (3)	
C3	0.1248 (8)	0.0915 (11)	0.3036 (10)	0.036 (3)	
C4	0.4859 (10)	0.1496 (12)	0.2468 (14)	0.054 (5)	
C5	0.4597 (8)	0.1355 (12)	0.0427 (13)	0.043 (4)	
C6	0.3904 (8)	−0.0134 (12)	0.1619 (10)	0.035 (3)	
C7	0.4075 (8)	0.3054 (13)	0.1500 (11)	0.041 (4)	
C8	0.3253 (7)	0.0368 (10)	0.4021 (9)	0.027 (3)	
C9	0.2741 (7)	0.0131 (11)	0.4646 (10)	0.033 (3)	
H9	0.2316	0.0561	0.4635	0.040*	
C10	0.2808 (9)	−0.0711 (11)	0.5302 (12)	0.050 (4)	
H10	0.2425	−0.0868	0.5713	0.060*	
C11	0.3421 (9)	−0.1317 (11)	0.5362 (11)	0.039 (4)	
H11	0.3477	−0.1889	0.5820	0.047*	
C12	0.3957 (9)	−0.1080 (13)	0.4742 (11)	0.046 (4)	
H12	0.4383	−0.1507	0.4770	0.055*	
C13	0.3897 (8)	−0.0239 (13)	0.4079 (10)	0.043 (4)	
H13	0.4281	−0.0076	0.3672	0.052*	
C14	0.2771 (8)	0.0159 (11)	−0.0427 (10)	0.035 (4)	
C15	0.3222 (8)	0.0223 (11)	−0.1190 (11)	0.042 (4)	
H15	0.3506	0.0853	−0.1277	0.050*	
C16	0.3259 (9)	−0.0641 (12)	−0.1833 (12)	0.056 (5)	
H16	0.3559	−0.0597	−0.2370	0.067*	
C17	0.2861 (10)	−0.1558 (12)	−0.1689 (13)	0.057 (5)	
H17	0.2890	−0.2152	−0.2122	0.069*	
C18	0.2416 (9)	−0.1620 (13)	−0.0915 (11)	0.046 (4)	
H18	0.2149	−0.2264	−0.0819	0.055*	
C19	0.2352 (8)	−0.0755 (10)	−0.0272 (10)	0.038 (4)	
H19	0.2035	−0.0788	0.0248	0.045*	
C20	0.1244 (6)	0.3322 (10)	0.1087 (10)	0.026 (3)	
H20	0.1192	0.2883	0.0522	0.031*	
C21	0.1002 (7)	0.4378 (11)	0.1022 (9)	0.029 (3)	
H21	0.0786	0.4650	0.0431	0.034*	
C22	0.1083 (7)	0.5032 (10)	0.1837 (10)	0.029 (3)	
C23	0.0838 (7)	0.6196 (11)	0.1818 (11)	0.034 (3)	
C24	0.0800 (8)	0.6762 (11)	0.0980 (12)	0.041 (4)	
H24	0.0946	0.6434	0.0398	0.049*	
C25	0.0545 (9)	0.7831 (12)	0.0961 (15)	0.055 (5)	
H25	0.0525	0.8218	0.0364	0.066*	
C26	0.0331 (9)	0.8316 (13)	0.1762 (16)	0.062 (5)	
H26	0.0148	0.9034	0.1733	0.074*	
C27	0.0379 (8)	0.7761 (11)	0.2643 (15)	0.051 (5)	
H27	0.0245	0.8100	0.3226	0.061*	
C28	0.0629 (7)	0.6686 (12)	0.2650 (13)	0.042 (4)	
H28	0.0653	0.6293	0.3243	0.051*	
C29	0.1396 (7)	0.4570 (11)	0.2687 (10)	0.035 (3)	
H29	0.1453	0.4986	0.3266	0.042*	
C30	0.1617 (7)	0.3514 (10)	0.2676 (10)	0.029 (3)	
H30	0.1833	0.3225	0.3260	0.035*	

### Atomic displacement parameters (Å^2^)

**Table:**

	*U*^11^	*U*^22^	*U*^33^	*U*^12^	*U*^13^	*U*^23^
Re1	0.0206 (3)	0.0269 (3)	0.0314 (3)	0.0006 (2)	−0.0005 (2)	−0.0038 (2)
Re2	0.0181 (3)	0.0254 (3)	0.0274 (3)	0.0002 (2)	−0.0063 (2)	0.0062 (2)
Te1	0.0187 (5)	0.0269 (4)	0.0250 (4)	0.0050 (3)	−0.0060 (4)	−0.0009 (3)
Te2	0.0294 (5)	0.0198 (4)	0.0231 (4)	0.0002 (3)	−0.0042 (4)	−0.0009 (3)
O1	0.056 (8)	0.029 (6)	0.077 (8)	0.001 (5)	−0.014 (7)	0.017 (5)
O2	0.053 (7)	0.054 (7)	0.065 (7)	−0.020 (6)	−0.042 (6)	0.011 (6)
O3	0.028 (6)	0.098 (10)	0.052 (7)	0.000 (6)	−0.002 (5)	0.028 (7)
O4	0.040 (8)	0.094 (10)	0.074 (9)	0.000 (7)	−0.033 (7)	0.002 (7)
O5	0.068 (9)	0.058 (8)	0.062 (8)	0.001 (6)	0.032 (7)	−0.010 (6)
O6	0.048 (7)	0.024 (6)	0.078 (8)	0.000 (5)	0.013 (6)	0.001 (5)
O7	0.069 (9)	0.027 (6)	0.070 (8)	−0.012 (5)	0.000 (7)	−0.003 (5)
N1	0.028 (6)	0.017 (5)	0.035 (6)	0.001 (5)	−0.007 (5)	0.002 (5)
C1	0.027 (8)	0.038 (9)	0.044 (9)	−0.007 (6)	−0.008 (7)	0.018 (7)
C2	0.040 (9)	0.020 (7)	0.044 (8)	−0.008 (6)	−0.011 (7)	0.020 (6)
C3	0.036 (3)	0.037 (3)	0.037 (3)	0.0004 (10)	0.0015 (10)	0.0002 (10)
C4	0.052 (12)	0.035 (9)	0.072 (12)	−0.001 (8)	−0.015 (10)	−0.002 (8)
C5	0.024 (8)	0.039 (9)	0.065 (11)	−0.003 (6)	−0.012 (8)	−0.007 (8)
C6	0.030 (9)	0.040 (9)	0.034 (8)	−0.005 (6)	−0.006 (7)	−0.004 (7)
C7	0.023 (8)	0.051 (10)	0.047 (9)	−0.010 (7)	−0.002 (7)	−0.022 (8)
C8	0.023 (7)	0.033 (7)	0.022 (6)	0.001 (6)	−0.013 (6)	−0.004 (6)
C9	0.009 (7)	0.036 (8)	0.054 (9)	0.007 (6)	0.001 (7)	0.005 (7)
C10	0.049 (10)	0.031 (8)	0.068 (11)	−0.014 (7)	−0.026 (9)	0.023 (8)
C11	0.050 (10)	0.030 (8)	0.035 (8)	0.000 (7)	−0.020 (8)	0.007 (6)
C12	0.044 (10)	0.054 (10)	0.038 (9)	0.021 (8)	−0.017 (8)	0.002 (7)
C13	0.036 (9)	0.058 (10)	0.037 (8)	0.009 (8)	0.009 (7)	0.002 (7)
C14	0.045 (10)	0.032 (8)	0.027 (7)	0.014 (7)	−0.015 (7)	−0.005 (6)
C15	0.041 (10)	0.029 (8)	0.054 (10)	0.002 (7)	−0.012 (8)	−0.003 (7)
C16	0.062 (12)	0.037 (9)	0.065 (11)	0.014 (8)	−0.027 (9)	−0.029 (8)
C17	0.070 (13)	0.036 (9)	0.064 (12)	0.018 (9)	−0.016 (10)	−0.026 (8)
C18	0.046 (4)	0.045 (4)	0.046 (4)	0.0000 (10)	0.0019 (10)	−0.0002 (10)
C19	0.054 (10)	0.022 (7)	0.034 (8)	−0.006 (7)	−0.028 (7)	0.006 (6)
C20	0.007 (6)	0.035 (7)	0.037 (8)	0.006 (5)	0.004 (6)	0.004 (6)
C21	0.025 (8)	0.041 (8)	0.020 (6)	−0.004 (6)	−0.002 (6)	0.002 (6)
C22	0.009 (7)	0.024 (6)	0.054 (9)	0.006 (5)	0.001 (6)	0.002 (6)
C23	0.011 (7)	0.043 (8)	0.047 (9)	−0.005 (6)	−0.001 (6)	0.000 (7)
C24	0.021 (8)	0.039 (8)	0.063 (10)	0.007 (6)	0.004 (7)	0.012 (8)
C25	0.040 (10)	0.035 (9)	0.092 (14)	0.002 (8)	0.019 (10)	0.013 (9)
C26	0.033 (10)	0.035 (9)	0.119 (17)	0.011 (7)	0.022 (11)	0.016 (11)
C27	0.030 (9)	0.027 (8)	0.097 (14)	0.003 (7)	0.012 (9)	−0.020 (9)
C28	0.013 (7)	0.049 (9)	0.066 (11)	−0.010 (6)	0.007 (7)	0.005 (8)
C29	0.021 (7)	0.037 (8)	0.046 (8)	0.010 (6)	−0.009 (7)	−0.006 (7)
C30	0.016 (7)	0.030 (7)	0.040 (8)	0.002 (5)	−0.002 (6)	−0.004 (6)

### Geometric parameters (Å, °)

**Table:**

Re1—C5	1.935 (18)	C13—H13	0.9500
Re1—C4	1.951 (18)	C14—C15	1.38 (2)
Re1—C7	1.951 (16)	C14—C19	1.39 (2)
Re1—C6	2.007 (15)	C15—C16	1.391 (19)
Re1—Te2	2.7832 (11)	C15—H15	0.9500
Re1—Te1	2.7872 (10)	C16—C17	1.37 (2)
Re2—C3	1.867 (15)	C16—H16	0.9500
Re2—C1	1.894 (15)	C17—C18	1.38 (2)
Re2—C2	1.906 (14)	C17—H17	0.9500
Re2—N1	2.218 (10)	C18—C19	1.40 (2)
Re2—Te2	2.7799 (11)	C18—H18	0.9500
Re2—Te1	2.7986 (10)	C19—H19	0.9500
Te1—C8	2.120 (13)	C20—C21	1.382 (18)
Te2—C14	2.145 (13)	C20—H20	0.9500
O1—C1	1.164 (16)	C21—C22	1.385 (18)
O2—C2	1.149 (16)	C21—H21	0.9500
O3—C3	1.212 (17)	C22—C29	1.402 (18)
O4—C4	1.117 (19)	C22—C23	1.509 (18)
O5—C5	1.175 (19)	C23—C24	1.35 (2)
O6—C6	1.136 (16)	C23—C28	1.37 (2)
O7—C7	1.152 (18)	C24—C25	1.40 (2)
N1—C30	1.325 (16)	C24—H24	0.9500
N1—C20	1.365 (16)	C25—C26	1.33 (2)
C8—C9	1.346 (18)	C25—H25	0.9500
C8—C13	1.410 (19)	C26—C27	1.39 (2)
C9—C10	1.380 (18)	C26—H26	0.9500
C9—H9	0.9500	C27—C28	1.41 (2)
C10—C11	1.36 (2)	C27—H27	0.9500
C10—H10	0.9500	C28—H28	0.9500
C11—C12	1.37 (2)	C29—C30	1.369 (18)
C11—H11	0.9500	C29—H29	0.9500
C12—C13	1.38 (2)	C30—H30	0.9500
C12—H12	0.9500		
			
C5—Re1—C4	93.7 (7)	C11—C12—C13	122.3 (14)
C5—Re1—C7	90.9 (6)	C11—C12—H12	118.9
C4—Re1—C7	88.4 (6)	C13—C12—H12	118.9
C5—Re1—C6	91.3 (6)	C12—C13—C8	118.4 (14)
C4—Re1—C6	93.8 (6)	C12—C13—H13	120.8
C7—Re1—C6	176.7 (6)	C8—C13—H13	120.8
C5—Re1—Te2	94.3 (4)	C15—C14—C19	121.9 (13)
C4—Re1—Te2	171.7 (6)	C15—C14—Te2	116.2 (11)
C7—Re1—Te2	89.0 (4)	C19—C14—Te2	121.9 (11)
C6—Re1—Te2	88.5 (4)	C14—C15—C16	119.5 (15)
C5—Re1—Te1	174.8 (4)	C14—C15—H15	120.3
C4—Re1—Te1	91.4 (6)	C16—C15—H15	120.3
C7—Re1—Te1	88.2 (4)	C17—C16—C15	119.9 (17)
C6—Re1—Te1	89.3 (4)	C17—C16—H16	120.1
Te2—Re1—Te1	80.57 (3)	C15—C16—H16	120.1
C3—Re2—C1	87.0 (6)	C16—C17—C18	120.3 (15)
C3—Re2—C2	93.2 (6)	C16—C17—H17	119.9
C1—Re2—C2	91.8 (6)	C18—C17—H17	119.9
C3—Re2—N1	91.6 (5)	C17—C18—C19	121.3 (15)
C1—Re2—N1	176.3 (5)	C17—C18—H18	119.4
C2—Re2—N1	91.7 (5)	C19—C18—H18	119.4
C3—Re2—Te2	175.5 (4)	C14—C19—C18	117.2 (15)
C1—Re2—Te2	96.2 (4)	C14—C19—H19	121.4
C2—Re2—Te2	89.8 (4)	C18—C19—H19	121.4
N1—Re2—Te2	85.1 (3)	N1—C20—C21	124.3 (12)
C3—Re2—Te1	96.5 (4)	N1—C20—H20	117.8
C1—Re2—Te1	89.3 (4)	C21—C20—H20	117.8
C2—Re2—Te1	170.3 (4)	C20—C21—C22	118.5 (12)
N1—Re2—Te1	87.5 (3)	C20—C21—H21	120.8
Te2—Re2—Te1	80.42 (3)	C22—C21—H21	120.8
C8—Te1—Re1	108.3 (4)	C21—C22—C29	117.6 (12)
C8—Te1—Re2	103.4 (3)	C21—C22—C23	121.5 (12)
Re1—Te1—Re2	96.67 (3)	C29—C22—C23	120.9 (12)
C14—Te2—Re2	108.3 (4)	C24—C23—C28	118.8 (14)
C14—Te2—Re1	102.0 (4)	C24—C23—C22	120.8 (14)
Re2—Te2—Re1	97.20 (3)	C28—C23—C22	120.4 (14)
C30—N1—C20	115.4 (11)	C23—C24—C25	120.6 (16)
C30—N1—Re2	123.0 (8)	C23—C24—H24	119.7
C20—N1—Re2	121.3 (8)	C25—C24—H24	119.7
O1—C1—Re2	177.8 (14)	C26—C25—C24	121.4 (17)
O2—C2—Re2	179.7 (16)	C26—C25—H25	119.3
O3—C3—Re2	177.2 (13)	C24—C25—H25	119.3
O4—C4—Re1	177.6 (15)	C25—C26—C27	119.4 (15)
O5—C5—Re1	177.4 (14)	C25—C26—H26	120.3
O6—C6—Re1	177.6 (13)	C27—C26—H26	120.3
O7—C7—Re1	176.7 (13)	C26—C27—C28	118.6 (16)
C9—C8—C13	118.1 (12)	C26—C27—H27	120.7
C9—C8—Te1	118.8 (9)	C28—C27—H27	120.7
C13—C8—Te1	123.0 (10)	C23—C28—C27	121.3 (16)
C8—C9—C10	122.7 (13)	C23—C28—H28	119.4
C8—C9—H9	118.6	C27—C28—H28	119.4
C10—C9—H9	118.6	C30—C29—C22	119.4 (13)
C11—C10—C9	120.1 (16)	C30—C29—H29	120.3
C11—C10—H10	119.9	C22—C29—H29	120.3
C9—C10—H10	119.9	N1—C30—C29	124.8 (13)
C10—C11—C12	118.3 (14)	N1—C30—H30	117.6
C10—C11—H11	120.9	C29—C30—H30	117.6
C12—C11—H11	120.9		
			
C5—Re1—Te1—C8	132 (5)	C7—Re1—C4—O4	19 (44)
C4—Re1—Te1—C8	−58.8 (6)	C6—Re1—C4—O4	−158 (44)
C7—Re1—Te1—C8	−147.2 (5)	Te2—Re1—C4—O4	−53 (46)
C6—Re1—Te1—C8	35.0 (5)	Te1—Re1—C4—O4	−69 (44)
Te2—Re1—Te1—C8	123.5 (3)	C4—Re1—C5—O5	−110 (30)
C5—Re1—Te1—Re2	26 (5)	C7—Re1—C5—O5	−22 (30)
C4—Re1—Te1—Re2	−165.3 (5)	C6—Re1—C5—O5	156 (30)
C7—Re1—Te1—Re2	106.4 (4)	Te2—Re1—C5—O5	68 (30)
C6—Re1—Te1—Re2	−71.5 (4)	Te1—Re1—C5—O5	59 (32)
Te2—Re1—Te1—Re2	17.05 (3)	C5—Re1—C6—O6	−69 (33)
C3—Re2—Te1—C8	55.6 (6)	C4—Re1—C6—O6	−163 (33)
C1—Re2—Te1—C8	−31.3 (6)	C7—Re1—C6—O6	65 (38)
C2—Re2—Te1—C8	−128 (2)	Te2—Re1—C6—O6	25 (33)
N1—Re2—Te1—C8	146.9 (5)	Te1—Re1—C6—O6	106 (33)
Te2—Re2—Te1—C8	−127.7 (4)	C5—Re1—C7—O7	−101 (25)
C3—Re2—Te1—Re1	166.2 (4)	C4—Re1—C7—O7	−7(24)
C1—Re2—Te1—Re1	79.3 (4)	C6—Re1—C7—O7	125 (22)
C2—Re2—Te1—Re1	−17 (2)	Te2—Re1—C7—O7	165 (25)
N1—Re2—Te1—Re1	−102.5 (3)	Te1—Re1—C7—O7	85 (24)
Te2—Re2—Te1—Re1	−17.08 (3)	Re1—Te1—C8—C9	−162.0 (9)
C3—Re2—Te2—C14	169 (6)	Re2—Te1—C8—C9	−60.2 (10)
C1—Re2—Te2—C14	34.1 (6)	Re1—Te1—C8—C13	21.8 (12)
C2—Re2—Te2—C14	−57.7 (5)	Re2—Te1—C8—C13	123.6 (11)
N1—Re2—Te2—C14	−149.4 (5)	C13—C8—C9—C10	−3(2)
Te1—Re2—Te2—C14	122.3 (4)	Te1—C8—C9—C10	−179.6 (11)
C3—Re2—Te2—Re1	64 (6)	C8—C9—C10—C11	2(2)
C1—Re2—Te2—Re1	−71.1 (4)	C9—C10—C11—C12	−1(2)
C2—Re2—Te2—Re1	−162.9 (4)	C10—C11—C12—C13	1(2)
N1—Re2—Te2—Re1	105.4 (3)	C11—C12—C13—C8	−2(2)
Te1—Re2—Te2—Re1	17.13 (3)	C9—C8—C13—C12	3(2)
C5—Re1—Te2—C14	53.1 (6)	Te1—C8—C13—C12	179.1 (10)
C4—Re1—Te2—C14	−144 (3)	Re2—Te2—C14—C15	−177.0 (9)
C7—Re1—Te2—C14	144.0 (6)	Re1—Te2—C14—C15	−75.1 (10)
C6—Re1—Te2—C14	−38.1 (6)	Re2—Te2—C14—C19	5.7 (12)
Te1—Re1—Te2—C14	−127.7 (4)	Re1—Te2—C14—C19	107.6 (11)
C5—Re1—Te2—Re2	163.6 (4)	C19—C14—C15—C16	1(2)
C4—Re1—Te2—Re2	−34 (3)	Te2—C14—C15—C16	−176.7 (11)
C7—Re1—Te2—Re2	−105.5 (4)	C14—C15—C16—C17	−2(2)
C6—Re1—Te2—Re2	72.4 (4)	C15—C16—C17—C18	1(2)
Te1—Re1—Te2—Re2	−17.19 (3)	C16—C17—C18—C19	1(3)
C3—Re2—N1—C30	51.4 (11)	C15—C14—C19—C18	1(2)
C1—Re2—N1—C30	−16 (9)	Te2—C14—C19—C18	178.3 (10)
C2—Re2—N1—C30	144.6 (11)	C17—C18—C19—C14	−2(2)
Te2—Re2—N1—C30	−125.7 (10)	C30—N1—C20—C21	−0.4 (19)
Te1—Re2—N1—C30	−45.1 (10)	Re2—N1—C20—C21	173.8 (10)
C3—Re2—N1—C20	−122.5 (10)	N1—C20—C21—C22	1(2)
C1—Re2—N1—C20	170 (8)	C20—C21—C22—C29	−1.1 (18)
C2—Re2—N1—C20	−29.2 (11)	C20—C21—C22—C23	179.4 (12)
Te2—Re2—N1—C20	60.5 (9)	C21—C22—C23—C24	−26.1 (19)
Te1—Re2—N1—C20	141.1 (10)	C29—C22—C23—C24	154.5 (14)
C3—Re2—C1—O1	7(36)	C21—C22—C23—C28	151.8 (13)
C2—Re2—C1—O1	−86 (36)	C29—C22—C23—C28	−27.6 (19)
N1—Re2—C1—O1	75 (38)	C28—C23—C24—C25	0(2)
Te2—Re2—C1—O1	−176 (100)	C22—C23—C24—C25	177.5 (13)
Te1—Re2—C1—O1	104 (36)	C23—C24—C25—C26	0(2)
C3—Re2—C2—O2	58 (100)	C24—C25—C26—C27	2(3)
C1—Re2—C2—O2	145 (100)	C25—C26—C27—C28	−2(2)
N1—Re2—C2—O2	−34 (100)	C24—C23—C28—C27	0(2)
Te2—Re2—C2—O2	−119 (100)	C22—C23—C28—C27	−178.1 (13)
Te1—Re2—C2—O2	−119 (100)	C26—C27—C28—C23	1(2)
C1—Re2—C3—O3	−62 (27)	C21—C22—C29—C30	1.1 (19)
C2—Re2—C3—O3	30 (27)	C23—C22—C29—C30	−179.5 (12)
N1—Re2—C3—O3	122 (27)	C20—N1—C30—C29	0.4 (19)
Te2—Re2—C3—O3	163 (22)	Re2—N1—C30—C29	−173.7 (11)
Te1—Re2—C3—O3	−151 (27)	C22—C29—C30—N1	−1(2)
C5—Re1—C4—O4	110 (44)		

### Hydrogen-bond geometry (Å, °)

**Table:**

*D*—H···*A*	*D*—H	H···*A*	*D*···*A*	*D*—H···*A*
C16—H16···O4^i^	0.95	2.55	3.33 (2)	139
C11—H11···O4^ii^	0.95	2.71	3.160 (18)	110
C17—H17···O1^iii^	0.95	2.46	3.271 (19)	144
C26—H26···O2^iv^	0.95	2.71	3.34 (2)	124
C21—H21···O3^v^	0.95	2.45	3.200 (16)	136
C29—H29···Cg^vi^	0.95	2.79	3.378 (15)	121

Symmetry codes: (i) −*x*+1, −*y*, −*z*; (ii) −*x*+1, −*y*, −*z*+1; (iii) *x*, −*y*−1/2, *z*−1/2; (iv) *x*, *y*+1, *z*; (v) *x*, −*y*+1/2, *z*−1/2; (vi) *x*, −*y*+1/2, *z*+1/2.
